# Randomized controlled trial on improving pesticide label interpretation among farmers in Akkar Governorate, Lebanon: The impact of a whatsapp-delivered educational video

**DOI:** 10.1371/journal.pone.0331842

**Published:** 2025-09-18

**Authors:** Nisreen Hassan Akkouch, Jalal Halwani, Issam Shaarani, Fouad Ziadeh

**Affiliations:** 1 Doctoral School of Science and Technology, Lebanese University, Tripoli, Lebanon; 2 Faculty of Medicine, Beirut Arab University, Beirut, Lebanon; Canadian University Dubai, UNITED ARAB EMIRATES

## Abstract

**Background:**

Pesticide misuse poses significant health and environmental risks, particularly in low- and middle-income countries. In Lebanon, improper pesticide handling and a lack of understanding of pesticide labels among farmers are major concerns. This study evaluated the effectiveness of a WhatsApp-delivered educational video compared to traditional in-person educational sessions in improving farmers’ pesticide safety knowledge, label interpretation, and handling practices.

**Design:**

This study employed a parallel-group, three-arm randomized controlled trial (RCT) with three groups: a traditional educational session group, a WhatsApp-delivered educational video group, and a control group receiving no intervention.

**Setting/participants:**

The study was conducted in the coastal villages of Akkar Governorate, northern Lebanon, from June to August 2024. One hundred thirty- three agricultural farmers were recruited through systematic sampling from a list of registered farmers, based on their pesticide use and access to WhatsApp.

**Interventions:**

Participants were randomly assigned to one of three groups: the control group received no intervention; the traditional education group participated in an in-person training session focused on pesticide safety, health risks, and label interpretation; the digital video group received a 4-minute educational video via WhatsApp, covering the same topics as the traditional session.

**Main outcome measures:**

Primary outcomes included the ability to interpret Food and Agriculture Organization of the United Nations (FAO) pictograms, knowledge of pesticide health risks, and understanding the environmental impacts of pesticide misuse. Secondary outcomes assessed the use of personal protective equipment (PPE) and changes in pesticide handling practices.

**Results:**

The video-based group showed the most significant improvement in pesticide handling, knowledge, and awareness of health and environmental risks, outperforming the traditional education group. The control group showed minimal changes. The video-based intervention was especially effective in enhancing the interpretation of pesticide labels, including complex pictograms and color codes.

**Conclusion:**

The results demonstrate that WhatsApp-delivered video interventions are superior in improving pesticide safety knowledge and practices compared to traditional methods. This cost-effective, scalable approach provides a viable solution for disseminating agricultural safety education, particularly in resource-limited areas. The study highlights the potential of digital learning tools to reach a wide audience with fewer resources, contributing to safer pesticide use and reducing health and environmental risks.

**Trial registration:**

International Standard Randomised Controlled Trial Number ISRCTN12809193

## Introduction

Pesticides have greatly boosted agricultural productivity, helping farmers achieve consistent crop yields worldwide [1]. However, pesticide exposure remains a serious public health concern, particularly in low- and middle-income countries (LMICs). In these regions, improper pesticide handling, insufficient farmer education, and weak regulatory oversight exacerbate the risks [2,3]. In Lebanon, agriculture plays a central role in rural livelihoods, but the lack of standardized pesticide safety training has led to widespread misuse, causing environmental damage and health risks [2]. Studies have shown that pesticide contamination in water resources and high pesticide residues in food products are common in areas like the Bekaa Valley and Akkar [4–6]. These findings highlight the pressing need to address pesticide misuse and its harmful effects on health and the environment.

A recent cross-sectional study conducted in the Akkar region found that most farmers lacked sufficient knowledge about pesticide safety, with only a small percentage following proper safety practices [7]. The study also showed that many farmers did not read pesticide labels, often relying instead on personal experience or advice from family members and pesticide sellers. These gaps in knowledge and practices highlight the need for effective educational programs to improve farmers’ understanding of pesticide safety. The study specifically pointed out that pesticide labels are often misunderstood, particularly in areas like Akkar, where low literacy rates make it difficult for farmers to interpret the labels properly [7]. These findings reinforce the importance of targeted interventions to address these knowledge gaps and encourage safer pesticide use in the region.

Pesticide exposure can result in severe health effects, ranging from acute symptoms like headaches, dizziness, and nausea to long-term conditions such as cancer, neurological disorders, and endocrine diseases [8,9]. Many Lebanese farmers misuse pesticides due to their limited knowledge of safe handling practices and the lack of formal training [10]. Pesticide labels, which should provide essential safety information, are often misunderstood, especially in areas like Akkar, where low literacy rates hinder farmers’ ability to interpret these labels [11,12]. This gap underscores the need for alternative education methods, such as visual or digital tools, to improve understanding and mitigate the risks.

In response to this, the FAO introduced pictograms and color-coded labels in 1988 to assist illiterate farmers in understanding safety instructions [13,14]. While these efforts aimed to improve safety, misinterpretation of pesticide labels continues to pose significant risks [11]. Despite these challenges, proper understanding of pesticide labels is essential to prevent health issues, food contamination, and environmental harm [15]. This study seeks to address this knowledge gap by exploring the effectiveness of a WhatsApp-delivered educational video on pesticide safety, comparing it to traditional in-person training. We aim to determine whether the video-based approach can improve farmers’ knowledge and practices, including their ability to interpret pesticide labels (color codes and pictograms).

The key question guiding this research is: How does a WhatsApp-delivered educational video compare to traditional in-person sessions in improving Lebanese farmers’ knowledge and practices regarding pesticide safety, health risks, and the interpretation of pesticide labels?

By evaluating this digital learning platform, the study contributes to the growing body of literature on digital interventions in agriculture. It also highlights a scalable and cost-effective solution to improve pesticide safety knowledge among Lebanese farmers, a strategy that could potentially be applied to other low-resource settings. Given the widespread use of **WhatsApp in Lebanon**, this platform offers a practical and scalable solution to reach dispersed farming communities with minimal logistical support [16]. The success of similar digital interventions, such as SMS-based education in Uganda [17] and video-mediated learning in Bangladesh [18], demonstrates the potential of digital tools to enhance farmers’ understanding and improve agricultural practices.

## Methodology

### Study design

This study is a parallel-group, three-arm, randomized controlled trial (RCT) conducted from June to August 2024 among agricultural farmers in the coastal villages of Akkar, northern Lebanon. The recruitment process occurred from June 10 to June 25, 2024. Participants were randomly assigned to one of three groups: the traditional educational session group (TESG), the digital video-based learning group (DVBLG), and the control group (CG). A total of 162 farmers were selected through systematic sampling from a list of 418 farmers registered in the municipality data to determine their current pesticide use and willingness to participate in the study. One hundred thirty- three farmers agreed to join and completed the baseline assessment through face-to-face interviews. The baseline focused on demographics, knowledge, practices, and understanding of pesticide labels (color codes and pictograms). Afterward, participants were randomly assigned to the three groups.

### Study area

The study was conducted in three coastal villages of Akkar Governorate, northern Lebanon, where agriculture is the primary livelihood and pesticide use is common due to the region’s climate and crop types. Akkar is known for pesticide contamination in groundwater and a lack of potable water [4,19].

### Participants

This study included 133 farmers selected from a list of 418 registered in Tal Hayat municipality and surrounding villages in Akkar. Using systematic sampling, every third farmer was contacted to assess eligibility and willingness to participate. Inclusion criteria required participants to be 18 years or older, actively engaged in agriculture, using chemical pesticides, and having access to WhatsApp. 150 eligible farmers were identified, with 133 attending the baseline assessment.

Participation was voluntary, and farmers could withdraw at any time. Attrition mainly occurred due to scheduling conflicts or personal reasons, with no significant demographic differences between those who withdrew and those who remained, suggesting random attrition.

Confidentiality: Participants' phone numbers were used as unique identifiers instead of names to ensure confidentiality and facilitate follow- up. Access to this data was restricted to authorized personnel, and all information was securely stored.

### Randomization

Participants were randomly assigned to one of the three groups using Excel’s **RAND function** to generate random numbers for each participant. The participants were then assigned to groups based on these numbers. Randomization ensured equal distribution across groups.

### Implementation

The implementation of the intervention was carried out as follows:

- **Control Group (CG):**

This group (n = 35) did not receive any intervention and served as the baseline for comparison. They were only involved in the baseline and follow-up assessments. Despite the initial randomization yielding 44 participants, nine farmers did not continue to the post-assessment. All participants in this group received transportation fees as an incentive to encourage participation.

- **Digital Video-Based Learning Group (DVBLG):**

This group (n = 34) initially included 45 participants; however, 11 farmers did not attend the session and the post-intervention assessment. Participants in this group watched a 4-minute educational video on pesticide safety via WhatsApp, featuring visual aids and a voice-over in Arabic to ensure clarity. The video covered safety practices, proper pesticide label interpretation, and understanding pictograms and color codes. Participants were able to view the video again as needed. Post-intervention, participants completed an assessment the next day, and transportation fees were provided as an incentive.

- **Traditional Educational Session Group (TESG):**

This group (n = 36) originally included 44 participants; however, 8 farmers decide to discontinue. Participants attended an in-person educational session led by the main researcher and supported by a plant protection specialist and environmental health professor. The session mirrored the video’s content, using PowerPoint presentations and group discussions. The session was conducted at the Safadi Foundation’s conference room in Der Dalloum, Akkar, and included incentives such as transportation fees and breakfast. Participants shared their experiences, which enhanced the learning experience.

### Interventions content

1. Traditional interventional session content: PowerPoint slides were used to educate farmers. The slides cover information related to the chemical pesticide definition, use, benefits, side effects on health, environmental impact (an image was used to illustrate how the pesticide can spread and pollute the environment: soil, groundwater, and air), occupational exposure, best handling techniques such as PPE use, and pesticide label understanding (14 pictograms and 4 color codes).
2. The educational video was approximately 4 minutes long and covered the same topics as the educational session. It included visual aids, explanations about good practices and misuse, a voice-over in Arabic, and sound effects to engage the farmers’ attention.

### Assessment tool

The assessment tool was created based on a thorough review of the literature in Lebanon [20] and relevant international literature [12–14,21,22]. The questionnaire consisted of four sections:

1. **Demographics:** Age, gender, years of experience, and educational level.
2. **Pesticide Practices:** Questions on pesticide safety measures, pesticide handling, PPE use, and past pesticide toxicity.
3. **Knowledge:** Focused on health impacts, environmental effects, and PPE importance. Likert scale questions were used in this section.
4. **Pesticide Label Understanding:** Focused on understanding color codes and pictograms on pesticide labels. Open-ended questions assessed the farmers’ ability to interpret these visual cues.

For statistical analysis, scores were assigned to the responses across the questionnaire to create continuous data. This allowed for the use of statistical tests such as the paired t-test to compare pre- and post-intervention outcomes.

The questionnaire was piloted with eight farmers, and based on their feedback, we made a few adjustments to ensure clarity and relevance. To further assess reliability, a test-retest procedure was conducted one week later with the same farmers, and their responses were manually compared to ensure consistency over time. The Cronbach’s Alpha values for the posttest data were as follows:

- Pesticide Handling: 0.69 (20 items)
- Knowledge: 0.86 (15 items)
- Awareness/Perception: 0.76 (5 items)
- Safety Labels and Instructions: 0.82 (20 items)

The overall Cronbach’s Alpha for the entire questionnaire was 0.90 (60 items), indicating strong internal consistency.

The questionnaire was translated from English to Arabic and administered through face-to-face interviews by trained surveyors proficient in Arabic, ensuring effective communication with participants. All data were entered using the Google Docs platform [23].

### Data collection

#### Baseline asessment.

The baseline assessment was conducted face-to-face at the Safadi Foundation conference room in Der Dalloum, Akkar. Two trained surveyors, familiar with the region and the study design, conducted the interviews to minimize errors and ensure consistent data collection. The surveyors were trained on ethical considerations, interview techniques, and questionnaire administration. The questionnaire was filled out in real-time using Google Docs, which allowed for immediate review and correction, reducing data entry errors.

#### Follow-up assessment.

The follow-up assessment took place after the interventions were completed, with the control group assessed first, followed by the traditional educational session group and finally the video-based group. This sequential order was chosen to reduce the potential for contamination between groups (e.g., video group participants sharing the video with others). This procedure helped maintain the integrity of the intervention effects.

#### Attrition and response rate.

The follow-up participation rate was high, with 133 farmers attending the baseline. However, 28 farmers dropped out primarily due to scheduling conflicts or voluntary withdrawal. Despite attrition, the final analytical sample consisted of 105 participants, maintaining sufficient statistical power.

#### Surveyor training.

Surveyors were trained in ethical data collection, informed consent procedures, confidentiality, and using the assessment tool. The training also emphasized being friendly and respectful with the farmers, ensuring they felt comfortable during the interviews. This was essential for building trust and reducing bias in responses

### Main outcome measures

The primary outcomes measured in this study included farmers’ ability to correctly identify and interpret FAO pictograms on pesticide labels, their knowledge regarding the health risks associated with pesticide use, and their understanding of the environmental consequences of pesticide misuse. Additionally, the study assessed farmers’ awareness of the importance of personal protective equipment (PPE) usage and reported changes in pesticide practices and PPE compliance before and after the educational interventions. These measures aimed to evaluate the effectiveness of the educational interventions (traditional vs innovative) in enhancing farmers’ knowledge and practices regarding pesticide safety.

### Sample size calculation

We calculated the sample size based on continuous outcome measures. We used effect size estimates from Damalas & Koutroubas (2014) to guide this recalculation [24]. In their study, the “beliefs” domain was identified as the most conservative measure, with trained farmers scoring a mean of 7.14 compared to 3.84 in untrained farmers, resulting in a Cohen’s d of 0.575. This was converted to an ANOVA-compatible effect size (f) using the standard formula (f = d/√2), yielding f ≈ 0.407. With a significance level (α) of 0.05, power (1 − β) of 0.95, and three groups, the analysis indicated a minimum total sample size of 84 participants (28 per group) to detect statistically significant differences. To account for potential attrition and ensure adequate statistical power, we aimed to recruit 45 participants per group (total 135 participants). This decision was based on our prior experience in the Akkar region, where we typically observe a response rate of about 2/3 of contacted farmers. Although some participants dropped out due to scheduling conflicts or voluntary withdrawal, the final analytical sample of 105 participants was still sufficient to achieve 95% statistical power for detecting significant differences in the outcome measures. Initially, we recruited 133 participants using systematic sampling. The final distribution of participants across the three groups was 45 participants in the DVBLG, and 44 participants in the CG and TESG. After attrition, the final number of participants that completed the study was 34 in the DVBLG, 35 in the CG, and 36 in the TESG. Thus, the final analytical sample consisted of 105 participants, which exceeded the minimum requirement for achieving 95% statistical power.

### Ethical approval and registration

Ethical approval was obtained from The Doctoral School of Science and Technology at the Lebanese University (reference number CE-EDST-6–2024). Initially planned as a quasi-experimental design, we later switched to an RCT approach to enhance scientific rigor. Although prospective trial registration was not completed before participant recruitment, the trial was retrospectively registered in the International Standard Randomised Controlled Trial Number (ISRCTN12809193) to ensure transparency.

### Statistical analysis

The data were analyzed using the Statistical Package for the Social Sciences (SPSS, version 25). Descriptive statistics were used to summarize participant demographics and scores. Paired t-tests were used to compare pre- and post-intervention scores within groups, and ANOVA with post hoc tests was used to compare differences between groups. Statistical significance was set at **p < 0.05** for all analyses.

## Results

This Randomized Controlled Trial was conducted between February and August 2024. One hundred thirty-three farmers from three villages in Akkar Valley were recruited through systematic sampling from a farmers’ registration at the principal city of these villages. A total of 133 participants were enrolled, but due to scheduling conflicts and personal reasons, 28 participants (22%) did not attend the follow-up assessments. As a result, the final analytical sample consisted of 105 participants: 34 in the DVBLG, 35 in the CG, and 36 in the TESG.

**Fig 1:**
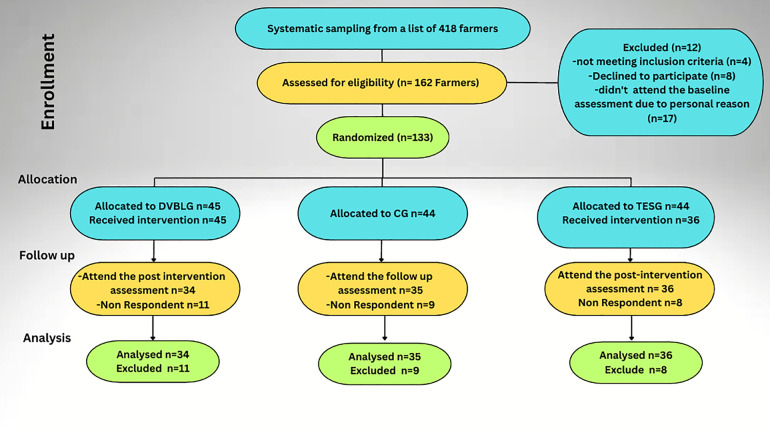
The flow of participants through each stage of the randomized trial.

Fig 1 describes the flow of participants through each stage of the study. The study participants’ follow-up rate was 78% (105/133). We used per-protocol outcome analyses; only participants who attended the interventions and presented for the follow-up assessment were included.

**Demographic Characteristics** Table 1 presents the baseline characteristics of the participants across the three study groups. No statistically significant differences were observed among the groups at baseline in terms of age, years of farming experience, gender, education level, receipt of pesticide safety training in the past two years, site of agricultural practice, or prior episodes of pesticide intoxication. The sample was predominantly male, with most participants having long farming experience. However, the majority had not received any pesticide safety training in the last two years.

**Table 1:** Baseline Characteristics of the Study Groups.

Characteristic	Control (N = 35)	Traditional education (N = 36)	Video-based education (N = 34)	p-value
Age in years (mean ± SD)	(46.1 ± 13.88)	(45.8 ± 13.60)	(45.1 ± 12.06)	0.934
Years of experience (mean ± SD)	(15.6 ± 9.056)	(16.9 ± 10.642)	(15.6 ± 9.829)	0.798
Gender				0.345
Male – n (%)	29(82.9%)	31(86.1%)	32(94.1%)	
Female – n (%)	6(17.1%)	5(13.9%)	2(5.9%)	
Educational level				0.846
Illeterate – n (%)	0(0.0%)	1(2.8%)	1(2.9%)	
School – n (%)	32(91.7%)	30(83.3%)	29(85.3%)	
University – n (%)	3(8.3%)	5(13.9%)	4(11.8%)	
Pesticide safety training in the last 2 years				0.244
Received training – n (%)	8(22.9%)	10(27.8%)	4(11.8%)	
Did not receive training – n (%)	27(77.1%)	26(72.2%)	30(88.2%)	
Site of practicing agriculture				
Open fields – n (%)	20(57.1%)	20(55.6%)	19(55.9%)	0.267
Green houses – n (%)	10(28.6%)	6(16.7%)	4(11.8%)	
Both – n (%)	5(14.3%)	10(27.8%)	11(32.4%)	
Previous episodes of pesticides intoxication				0.608
Present – n (%)	8(22.9%)	7(19.4%)	5(14.7%)	
Absent – n (%)	27(77.1%)	28(77.8%)	27(79.8%)	
Unsure – n (%)	0.00%	1(2.8%)	2(5.9%)	

**Impact of Educational Interventions** Table 2 summarizes the pre- and post-intervention scores across the three study groups for four key outcome domains: pesticide handling, knowledge, awareness/perception, and understanding of safety labels and instructions. A one-way ANOVA was used to assess between-group differences at both pre- and post-intervention stages, while paired t-tests assessed within-group changes.

**Table 2:** Pre- and Post-Intervention Scores of the Study Groups.

Construct	Group	Pre Score (Mean ± SD)	Pre Score p-value	Post Score (Mean ± SD)	Post Score p-value	Pre vs Post p-value
Pesticide Handling	Control	37.71 ± 18.04	0.124	35.00 ± 18.59	0.004	0.537
	Traditional	44.44 ± 16.03		40.97 ± 11.52		0.295
	Video-based	37.94 ± 12.00		46.76 ± 11.67		**0.003**
Knowledge	Control	65.20 ± 17.33	0.923	64.22 ± 18.19	0.002	0.821
	Traditional	66.33 ± 16.57		77.67 ± 18.92		0.009
	Video-based	64.71 ± 18.64		77.53 ± 15.64		**0.003**
Awareness/ Perception	Control	39.43 ± 21.82	0.345	41.43 ± 20.60	<0.001	0.695
	Traditional	46.94 ± 22.40		60.83 ± 19.62		**0.007**
	Video-based	44.41 ± 21.49		61.47 ± 22.85		**0.002**
Safety Labels & Instructions	Control	48.11 ± 15.29	0.667	50.86 ± 13.97	<0.001	0.444
	Traditional	45.99 ± 17.62		64.56 ± 20.29		**<0.001**
	Video-based	49.18 ± 13.49		72.12 ± 16.55		**<0.001**

*Note: p-values represent between-group comparisons at pre and post stages (ANOVA), and within-group pre-post changes (paired t-tests).

**Pesticide Handling** The video-based education group demonstrated a significant increase in post-intervention scores, from 37.94 ± 12.00 to 46.76 ± 11.67 (p = 0.003). The traditional and the control group didn’t show a statistically significant improvement, p value were not significant for these two groups. Post hoc analysis as shown in Table 3 revealed that the video-based group performed significantly better than the control group **p < 0.001**, while the difference between video-based and traditional groups was not significant (p = 0.28).

**Table 3:** Post Hoc Pairwise Comparisons Between Groups (Post-Intervention Scores).

Construct	Comparison	Mean Difference (I–J)	Standard Error	p-value
Pesticide Handling	Control¹ vs. Traditional¹˙²	−6.67	3.38	0.15
	Control¹ vs. Video²	−12.08*	3.38	0.002
	Video² vs. Traditional¹˙²	+5.42	3.38	0.28
Knowledge	Control¹ vs. Traditional²	−13.39*	4.18	0.01
	Control¹ vs. Video²	−12.39*	4.18	0.01
	Traditional² vs. Video²	+1.00	4.18	0.97
Awareness	Control¹ vs. Traditional²	−19.44*	4.92	<0.001
	Control¹ vs. Video²	−19.72*	4.92	<0.001
	Video² vs. Traditional²	+0.28	4.92	1.00
Safety Labels	Control¹ vs. Traditional²	−13.22*	4.05	0.01
	Control¹ vs. Video²	−20.11*	4.05	<0.001
	Video² vs. Traditional²	+6.89	4.05	0.24

*Significant at p < 0.05. Superscripts: ¹Control, ²Video-based, ¹˙²Traditional. Post hoc tests were conducted using Tukey’s correction. Mean difference (I–J) represents the difference between the group listed first (I) and the group listed second (J). Negative values indicate that group J scored higher than group I.*

**Knowledge** Both the traditional and video-based education groups experienced significant improvements. The traditional group improved from 66.33 ± 16.57 to 77.67 ± 18.92 (p = 0.009), and the video-based group from 64.71 ± 18.64 to 77.53 ± 15.64 (p = 0.003). The control group did not show a significant change (p = 0.821). Post hoc analysis showed that both intervention groups outperformed the control group significantly (p = 0.01 for both comparisons), while the difference between traditional and video-based groups was not statistically significant (p = 0.97).

**Awareness and Perception** The video-based group showed the most improvement in awareness scores, increasing from 44.41 ± 21.49 to 61.47 ± 22.85 (p = 0.002). The traditional group also improved significantly (from 46.94 ± 22.40 to 60.83 ± 19.62, p = 0.007). The control group’s change was minimal and not statistically significant (p = 0.695). Post hoc analysis indicated that both traditional and video-based groups performed significantly better than the control group (p < 0.001 for both), with no significant difference between them (p = 1.00).

**Safety Labels and Instructions** Significant improvements were observed in both intervention groups. The traditional group improved from 45.99 ± 17.62 to 64.56 ± 20.29 (p < 0.001), and the video-based group improved from 49.18 ± 13.49 to 72.12 ± 16.55 (p < 0.001). The control group showed no meaningful change (p = 0.444). Post hoc analysis showed that the video-based group outperformed both the control group (p < 0.001) and the traditional group (p = 0.24), while the traditional group also performed significantly better than the control group (p = 0.01).

## Discussion

This study demonstrated the effectiveness of a video-based educational intervention in improving pesticide safety knowledge, handling practices, and understanding of safety labels among farmers in the Akkar region of Lebanon. The video-based group showed significant improvements across all measured domains, particularly in pesticide handling and safety label interpretation, compared to both the control and traditional education groups. These findings highlight the value of digital tools in agricultural education, especially in contexts where logistical and resource constraints limit the feasibility of traditional, in-person training methods.

**Fig 2:**
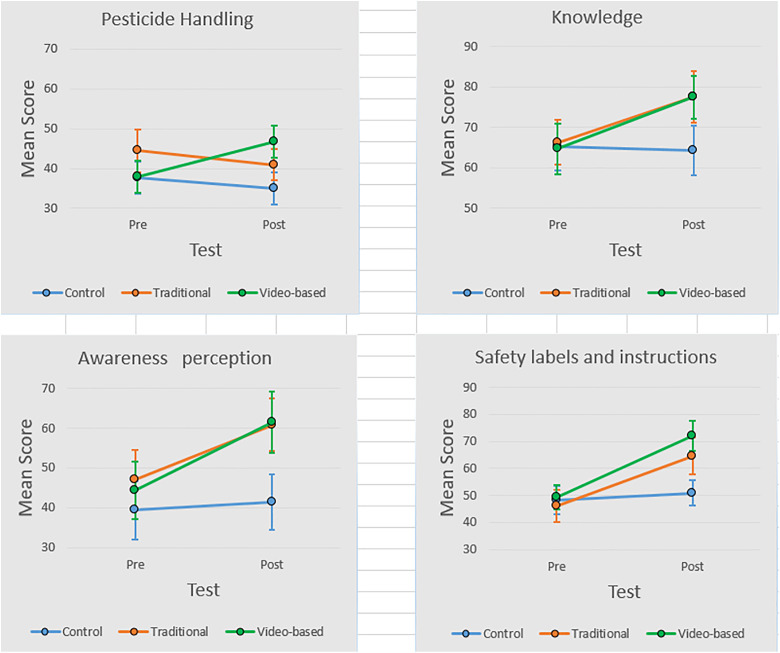
Comparison of Pesticide Safety Knowledge, Handling Practices, Awareness, and Label Interpretation Scores Among Control, Traditional, and Video-Based Educational Groups.

As shown in Fig 2, the video-based group experienced the most substantial improvement in scores across all domains, including pesticide handling, knowledge, awareness, and safety labels. The graph illustrates the significant increase in mean scores from pre- to post-intervention, particularly in pesticide handling and safety labels and awareness, which supports the positive impact of the video-based educational intervention.

Our results align with those of Chowdhury et al. (2010), who found that video-mediated learning was highly effective in promoting farmers’ understanding of botanical pesticide management in Bangladesh [18]. Similarly, our findings suggest that video-based learning significantly enhanced farmers’ ability to interpret pesticide labels and improve their handling practices. The visual and interactive nature of video likely contributed to higher engagement and knowledge retention compared to the traditional method.

These findings also align with those of Maddah et al. (2019) in Lebanon, where a community-based educational intervention successfully improved farmers’ knowledge, attitudes, and practices regarding pesticide safety [10]. While their study used in-person workshops, our video-based intervention showed similar improvements in knowledge and safety practices, with the added advantage of scalability. This scalability is a key strength of video-based interventions, as they can be distributed to a large audience at a lower cost compared to traditional training models that require significant and costly resources.

The SMS-based intervention used by Ssekkadde et al. (2021) in Uganda also illustrates the power of digital tools in agricultural extension services. This study demonstrated that SMS interventions, combined with traditional workshops, could significantly improve safe pesticide practices among Ugandan farmers [17]. A notable finding from their study, which aligns with ours, is the scalability of digital interventions. While their focus was on SMS text messages, our study highlights that video-based learning can also serve as a cost-effective and wide-reaching educational tool. The flexibility of videos allows farmers to access the material remotely, at their convenience, thus overcoming the challenges posed by geographic barriers or limited access to in-person training sessions. Using platforms like WhatsApp, educational videos can easily reach many farmers, who can then share them with others, all with little logistical effort.

While our educational video was in Arabic with clear visual aids, future interventions could benefit from addressing additional important subjects that farmers commonly face to ensure broader relevance and aligns with the farmers’ real-world challenges and enhances its impact.

While the video-based intervention proved effective, it is important to recognize the potential barriers to digital education, especially in rural areas with varying levels of digital literacy. In our study, we carefully selected farmers who were familiar with using mobile phones and had access to WhatsApp, ensuring that the intervention would reach those capable of engaging with the content. However, digital illiteracy remains a challenge in many low-resource settings, and some farmers may face difficulties accessing, understanding, or engaging with digital content [25,26]. As digital tools become an increasingly important part of educational interventions, it is essential to design content that is simple, engaging, and culturally appropriate, while also considering the varying levels of technological access and literacy among the target audience [27]. By addressing both digital illiteracy and cultural relevance, digital educational tools can become more accessible and effective for farmers in rural and underserved regions.

By adopting video learning, our study demonstrates the effectiveness of a low-cost, flexible educational tool that can significantly enhance farmers’ engagement and knowledge retention in a wide-reaching manner. As digital technologies continue to evolve, further research is needed to explore the combination of video learning with other digital platforms, such as mobile apps or SMS, to further expand the reach and impact of agricultural education interventions.

## Limitations

This study has several limitations. Due to time constraints, we were unable to assess long-term retention of knowledge. We used a per-protocol analysis instead of the preferred intention-to-treat approach to evaluate intervention efficacy under ideal conditions, which may limit the generalizability of findings to real-world settings. The study also excluded farmers without access to WhatsApp and those with literacy challenges, potentially introducing selection bias. Despite these limitations, the study provides valuable insights into the effectiveness of digital video-based education in improving pesticide safety practices among farmers.

## Conclusion

This study demonstrated the effectiveness of a WhatsApp-delivered educational video in enhancing pesticide label interpretation and safety practices among farmers in the Akkar Governorate, Lebanon. The video-based intervention was more effective than traditional in-person educational sessions in improving farmers’ knowledge, awareness, and practices regarding pesticide use. This suggests that digital video-based education can be a scalable and resource-efficient solution for disseminating critical agricultural safety information, particularly in low-resource settings. By leveraging widely accessible platforms like WhatsApp, such interventions can reach a larger audience, reduce costs, and ensure consistent, repeated exposure to safety messages, ultimately promoting safer pesticide handling practices and reducing the associated health and environmental risks.

## Supporting information

S1 File The questionnaire used in the baseline and post-intervention assessments. (PDF)

S2 File The randomized controlled trial (RCT) protocol. (DOCX)

S1 Checklist The CONSORT checklist. (DOC)

S1 Data The anonymized raw data used to generate the study results. These supplementary materials are essential for evaluating the study methodology and support the reproducibility and transparency of the research.
(CSV)
