# Supplementary material for: Randomized controlled trial on improving pesticide label interpretation among farmers in Akkar Governorate, Lebanon: The impact of a whatsapp-delivered educational video
Source: PLoS One. 2025 Sep 18;20(9):e0331842. doi: 10.1371/journal.pone.0331842 (PMC12445491; doi:10.1371/journal.pone.0331842)
Supplement: S1 File — (PDF) [file pone.0331842.s001.pdf]

Survey Form.

phone number (WhatsApp): ---------------------------------- Date:

Demographics:

Age: -----------------------

Gender: -----------------

Years of experience: --------------------

Educational Level: [Illiterate / Primary school / Intermediate / Secondary / University]

Pesticide Handling:

1. Have you received pesticide safety training during the last two years?

Yes No

2. Do you handle pesticides in agriculture? (Mixing, spraying, cleaning materials)

Yes No

3. Where do you practice agriculture? (Select one or more):

Open field Greenhouses Both

4. Have you ever experienced pesticide poisoning? (episode of pesticide intoxication)

Yes No Unsure

Safe Handling Practices: Please mark the PPE you use during pesticide handling (preparation and spraying).

**Preparation**	**Spraying**
Gloves	Gloves
Face mask (surgical or fabric scarf)	Face mask
Long sleeve	Long sleeve
Pants	Pants
Face shield	Face shield
Goggles	Goggles
hat	hat
Coverall (spray suit)	Coverall (spray suit)

Knowledge: **(Question 1. Each choice will take 4 points, and the total will be 40 points. Question 2. Each correct choice will get 3 points, and if he chooses the three correct choices will get 10 points. The total score for this part will be 50 points)**

1. Please rate the importance of each safety measure during pesticide handling, from 1 to 5 with 1 not important at all and 5 extremely important.

Wearing gloves:

Wearing long sleeves:

Wearing a mask:

Wearing a hat:

Wearing pants:

Wearing boots:

Wearing goggles:

Wearing face shield:

Avoid reentry after pesticide spraying:

Respecting preharvest interval:

2. What is the primary way pesticides enter a farmer's body during work? Select all that apply.
   - Through inhalation (breathing it in)
   - Through skin contact (dermal exposure)
   - Through swallowing (oral ingestion)
   - I'm not sure/ I don't know
   - Pesticides do not enter the body

Awareness or Perception: **this part consists of 10 questions, 2 points for strongly agree, 1 point for agree, and zero point for disagree, and strongly disagree. The total score will be 10 points)**

1. Do you believe that exposure to pesticides has a negative impact on human health?
   - Strongly agree
   - Agree
   - Disagree
   - Strongly disagree

2. Do you think that pesticides contaminate groundwater?
   - Strongly agree
   - Agree
   - Disagree
   - Strongly disagree

3. Do you think that pesticide spraying harms beneficial insects?
   - Strongly agree
   - Agree
   - Disagree
   - Strongly disagree

4. Do you think that pesticide spraying affects the soil quality?

- Strongly agree
- Agree
- Disagree
- Strongly disagree

5. Do you believe that pesticide use leads to air pollution?
- Strongly agree
- Agree
- Disagree
- Strongly disagree

Safety Labels and Instructions: (the total score for this part is 25 points)

1. How often do you read safety labels on pesticide containers before use? (3 points for “always,” 1 point for “sometimes,” and zero for “rarely or never.”

Always sometimes rarely, or never

2. Do you check the expiry date on the pesticide container before using it?

Always sometimes rarely, or never

3. Explain the meaning of each color label. (1 point for correct explanation, and zero for incorrect. Participants who could explain the four labels correctly will get 5 points.)

   - Red color ---
   - Yellow color ---
   - Blue color --
   - Green color --

4. Explain the meaning of each Pictogram. (14 points total, 1 point for every correct explanation).

Activity Pictograms

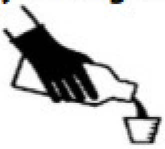

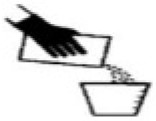

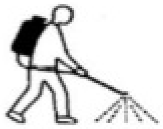

Storage and Warning Pictograms

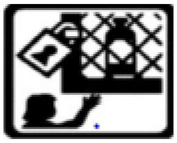

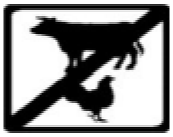

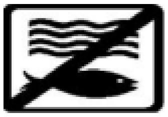

Advice Pictograms

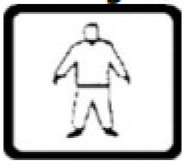

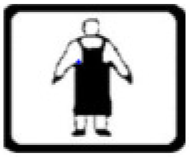

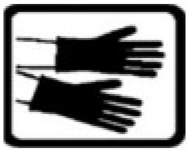

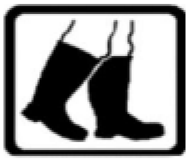

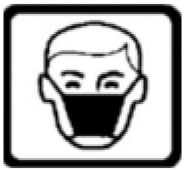

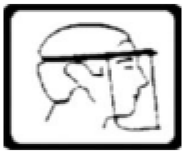

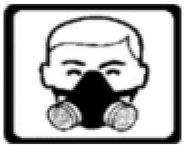

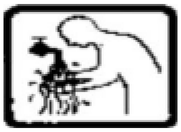

We used these questions to evaluate the educational video from the video group.

1. **Educational Video Content Evaluation.**
    - Was the information in the video presented clearly and easy to understand?

      ☐ Not clear

      ☐ Somewhat clear

      ☐ Very clear

2. **Practical Benefit:**
    - Did you find the video beneficial for understanding and applying safe pesticide use in your farming practices?

      ☐ Not Beneficial

      ☐ Somewhat beneficial

      ☐ Very beneficial

3. **Compared to Traditional Learning**
    - Do you find learning from this video more effective than attending traditional classes?

      ☐ Less effective

      ☐ About the same

      ☐ More effective

4. **Convenience:**
    - Do you find watching this video more convenient than attending educational sessions?

    ☐ Less convenient

    ☐ About the same

    ☐ More convenient
