# Supplementary material for: Randomized controlled trial on improving pesticide label interpretation among farmers in Akkar Governorate, Lebanon: The impact of a whatsapp-delivered educational video
Source: PLoS One. 2025 Sep 18;20(9):e0331842. doi: 10.1371/journal.pone.0331842 (PMC12445491; doi:10.1371/journal.pone.0331842)
Supplement: S2 File — (DOCX) [file pone.0331842.s002.docx]

**Protocol S1**

**Randomized Controlled Trial on Improving Pesticide Label Interpretation Among Farmers in Akkar Governorate, Lebanon: The Impact of a WhatsApp-delivered Educational Video.**

**Authors:** Nisreen Akkouch^1*^, Jalal Halwani^1^, Issam Shaarani^2^, Fouad Ziadeh^3^

**Affiliations:**

1. Lebanese University/ Doctoral School of Science and Technology
2. Beirut Arab University/ Faculty of Medicine.
3. Lebanese University/ Faculty of Public Health section 3.

**1. Introduction**

The use of plant protection products (PPPs) will continue to play a significant role in modern agriculture, significantly boosting productivity and allowing farmers to meet increasing global food demands (Damalas, 2009). However, while pesticides offer undeniable benefits, their misuse will remain a significant global public health concern, particularly in low- and middle-income countries (LMICs), where regulatory oversight is often limited (Abou Zeid et al., 2020; Handford et al., 2015). In Lebanon, agriculture is central to rural livelihoods. However, there is insufficient standardized training on pesticide safety, leading to widespread misuse and exposing the environment and human health to significant risks (Abou Zeid et al., 2020; Maddah et al., 2020).

Pesticide exposure has been linked to both acute and chronic health impacts, ranging from immediate symptoms like dizziness and nausea to long-term conditions such as cancer and neurological disorders (Mostafalou & Abdollahi, 2017; Nicolopoulou-Stamati et al., 2016). Despite these well-known risks, many farmers in rural Lebanon will continue to misuse pesticides due to insufficient knowledge about safe handling practices and inadequate access to formal education programs (Maddah et al., 2020). Moreover, the environmental consequences of pesticide misuse, such as water, soil, and air contamination, will continue to pose serious ecological risks (Gevao et al., 2022; Syafrudin et al., 2021; Tsai, 2013). Therefore, addressing this issue through effective educational interventions will be crucial for improving safety practices in farming communities.

Pesticide labels are the primary means manufacturers communicate crucial safety information to users, especially in LMICs (World Health Organization, 2022). However, the effectiveness of these labels will often be compromised by literacy challenges and a lack of understanding of the symbols and color codes they employ. For example, in Lebanon's rural regions like Akkar, where literacy rates are low, farmers will struggle to interpret these labels, thus elevating the risk of unsafe practices (Abou Ibrahim et al., 2023; Waichman et al., 2007). To bridge this knowledge gap, accessible and scalable educational methods, such as video-based interventions, will need to be implemented.

This study will evaluate the effectiveness of a WhatsApp-delivered educational video compared to traditional in-person educational session in improving pesticide label interpretation and safety practices among farmers in Akkar Governorate, Lebanon. Using widely accessible platforms like WhatsApp will allow disseminating important safety information to a larger audience with fewer resources. By leveraging the widespread use of digital technology in the region, this approach aims to provide a scalable solution to reach geographically dispersed and resource-limited farming communities (MHEIDLY et al., 2021). We hypothesize that the WhatsApp-delivered video will be as effective as traditional in-person education in improving farmers’ pesticide handling practices, knowledge, and understanding of label pictograms and color codes.

This study will fill a critical gap in the literature on educational interventions for pesticide safety in LMICs, particularly in Lebanon, where there have been limited studies on innovative training methods for farmers (Maddah et al., 2020; Mardigian et al., 2021). The findings will provide valuable insights into developing effective, resource-efficient interventions to improve pesticide safety, thus promoting both human health and environmental protection in farming communities.

**Hypothesis**

We hypothesize that a WhatsApp-delivered educational video will be as effective as a traditional in-person educational session in enhancing farmers' pesticide safety knowledge, practices, and interpretation of pictograms and color codes on pesticide labels.

**Study Objectives**

- **Primary Objective**: To assess the effectiveness of a WhatsApp-delivered educational video compared to a traditional in-person educational session in improving the interpretation of pesticide labels and safety practices among farmers in Akkar Governorate, Lebanon.
- **Secondary Objective**: To determine if the WhatsApp-delivered video improves the use of personal protective equipment (PPE) and safe pesticide-handling practices.

**Ethical Considerations**

This study will be conducted following ethical guidelines, with approval sought from the Institutional Review Board (IRB) of The Doctoral School of Science and Technology at the Lebanese University. The study will not commence until IRB approval is granted, and participants will be fully informed about the study's objectives, procedures, and their rights.

**4. Study Design**

This will be a parallel-group, three-arm, randomized controlled trial (RCT) designed to evaluate the impact of two educational interventions on farmers' knowledge and practices related to pesticide use. Similar content will be used in both the educational video and the educational session.

**Study Arms**:

1. **Control Group (CG)**: Farmers will receive no intervention.
2. **Traditional Educational Session Group (TESG)**: Farmers will attend an in-person educational session on pesticide safety, health impacts, and label interpretation.
3. **Digital Video-Based Learning Group (DVBLG)**: Farmers will receive a 4-minute educational video via WhatsApp containing the same content as the in-person session.

**Randomization**

Participants will be randomly assigned to one of three groups: the Control Group, the Digital Video-Based Learning Group (DVBLG), and the Traditional Educational Session Group (TESG). Each participant’s phone number will serve as their unique identifier, simplifying group allocation and follow-up communication without requiring participants to remember a specific code. To ensure a random allocation, we will use the RAND function in Microsoft Excel.

The process will be as follows:

1. Each participant’s phone number will be entered in a single column in Excel. The RAND function will generate a random number for each participant. These random numbers will then be used to sort the list of participants in ascending order, resulting in a randomized list.
2. Based on this sorted list, participants will be allocated sequentially to each of the three groups, ensuring equal distribution.
3. To facilitate communication, we will create a WhatsApp group for each team. We will use the group to invite the TESG to the educational session, send the educational video to the DVBLG, and provide them with instructions to watch it. Additionally, we will use the group to call them for the post-assessment.

This approach will ensure a transparent and unbiased randomization process, allowing us to assess intervention effectiveness across comparable groups.

**5. Participants**

**Inclusion Criteria**:

- Farmers aged 18 years and older.
- Engaged in agricultural activities in the coastal region of Akkar.
- Actively using chemical pesticides.
- Access to WhatsApp on their own or a family member's device.

**Exclusion Criteria**:

- Farmers who do not meet the inclusion criteria.

A total of 133 participants will be enrolled and randomized.

**Participants Consent**

All participants will be fully informed about the research objectives, procedures, potential risks, and benefits before enrolling in the study. The consent process will prioritize participant autonomy and comprehension to ensure ethical standards are upheld.

Consent Procedure:

1. Information Session: Each participant will receive a clear, accessible explanation of the study objectives, design, and procedures in Arabic, detailing any potential risks and anticipated benefits, including the educational value of the intervention. Participants will be informed that their participation is voluntary, and they may withdraw from the study at any point without consequence.
2. Consent Form: Participants will receive a written consent form outlining key study details. This form will require a signature or, for illiterate participants, a fingerprint to indicate their agreement to participate. Participants can also ask questions about the study and receive clarification.
3. Confidentiality and Anonymity: Participants will be assured of strict confidentiality, and unique phone numbers will be used as identifiers to protect personal data. Only authorized research personnel will access identifiable information; all data will be anonymized in reports and publications.
4. Voluntary Participation and Withdrawal: It will be made clear that participation is entirely voluntary, with no penalty for declining or withdrawing. Participants may choose to exit the study at any time without affecting any future interactions or support provided through the study.

**Participants recruitment:**

Participants for this study will be recruited from a list of 418 agricultural farmers provided by the municipalities of three nearby coastal villages in the Akkar Governorate, northern Lebanon. These farmers are registered in the municipal database, making them eligible for recruitment in the study due to their involvement in agriculture within this region. Systematic sampling will be used, whereby every third farmer on the list will be contacted to determine eligibility and interest in participating.

To qualify, participants must be at least 18 years old, engaged in agricultural activities within the coastal area, actively using chemical pesticides, and have access to WhatsApp on a personal or family member's device. Farmers meeting these criteria will be informed about the study’s objectives, procedures, and voluntary nature, emphasizing confidentiality and the right to withdraw at any time. Consent will be obtained through signatures or fingerprints, depending on literacy levels. Farmers who are not eligible or choose not to participate will be replaced by the next farmer on the list, and this process will continue until the required sample size of 150 participants is reached.

**6. Sample Size Calculation**

The sample size calculation for this randomized controlled trial will be based on prior data from a cross-sectional study conducted in the same region, which found that only 4% of farmers routinely read pesticide labels before preparing pesticide mixtures. To achieve a statistically meaningful improvement with our educational intervention, we aim to detect an increase in this behavior by 25%—raising it from 4% to 29%.

Using a significance level of 0.05 and a power of 80%, we estimate that each group (Digital Video-Based Learning Group, Traditional Educational Session Group, and Control Group) will require approximately 35 participants, totaling 105 participants across the three groups. However, we plan to recruit additional participants to account for potential attrition and ensure a robust final sample. The number of these additional participants will be determined based on observed participation and retention rates during the recruitment phase, allowing us to meet our target sample size despite potential dropouts. This approach will help maintain sufficient statistical power throughout the study, even if some participants cannot complete all stages.

7. **Description of the interventions.**

This randomized controlled trial will involve three distinct groups, each receiving different levels of intervention:

1. **Control Group (CG)**
   Participants in the control group will receive no educational intervention and will serve as a baseline to compare the effects of the other interventions. This group will only participate in baseline and follow-up assessments to measure changes in knowledge, practices, and understanding of pesticide labels without external educational support.
2. **Digital Video-Based Learning Group (DVBLG)**
   The participants in this group will receive a 4-minute educational video on pesticide safety distributed via WhatsApp. The video will cover essential topics on pesticide use, focusing on reading pesticide labels, understanding FAO pictograms, interpreting color codes, and practicing safe handling procedures, including using personal protective equipment (PPE). Visual aids and examples will be incorporated into the video to enhance comprehension, and the content will be presented in Arabic to ensure clarity and accessibility. The video will be available for rewatching, allowing flexible, self-paced learning. Participants in this group will receive a transportation fee as an incentive for participating.
3. **Traditional Educational Session Group (TESG)**
   Participants in the traditional educational session group will attend an in-person, interactive training session. This session, led by the primary researcher with support from a plant protection specialist and an environmental health professor, will use PowerPoint presentations and encourage group discussions. Topics covered will mirror those in the video, including pesticide safety, label interpretation, pictograms, color coding, and PPE usage. The face-to-face format will allow for immediate questions, personalized guidance, and opportunities for farmers to share their experiences. The session will take place in the Safadi Foundation’s conference room in Der Dalloum, Akkar, with participants receiving transportation fees and breakfast as incentives.

Each intervention is designed to improve the participants' knowledge, practices, and interpretation of pesticide safety labels, with the goal of assessing which format—digital or traditional—is more effective in a resource-limited setting. The interventions are specifically crafted to address language and literacy barriers, ensuring accessible and practical education for the farmers involved.

**8. Materials**

Questionnaire

The data for this study will be collected using a structured questionnaire that has been developed based on a thorough review of relevant literature on pesticide use and safety practices among agricultural communities in Lebanon and internationally (Abou Ibrahim et al., 2023; Dugger-Webster & LePrevost, 2018; Emery et al., 2015). The questionnaire will consist of four primary sections:

1. **Demographics**: This section will capture participants’ age, gender, years of farming experience, and education level.
2. **Pesticide Practices**: Questions will cover prior training on pesticide safety, pesticide handling practices, types of agricultural environments (open field or greenhouse), and the use of personal protective equipment (PPE). Most questions in this section will be closed-ended.
3. **Knowledge**: This section will measure the participants' awareness of the health risks associated with pesticide use, environmental hazards of pesticides, and the importance of PPE. Responses will be collected using Likert scale questions for a range of agreement or knowledge levels.
4. **Pesticide Label Understanding**: This section will assess participants’ ability to interpret pictograms and color codes on pesticide labels. Due to their complexity, open-ended questions will be used to gauge their comprehension.

The questionnaire will be administered face-to-face by trained surveyors proficient in Arabic to ensure clear communication and facilitate accurate data collection. The surveyors will record responses in real-time using Google Docs, which allows for immediate review to minimize data entry errors. To ensure accessibility for all participants, particularly those with low literacy levels, surveyors will read questions aloud and provide clarification as needed.

**Educational Session**

The traditional educational session (TESG) will be delivered in person and will cover critical topics related to pesticide safety. The principal researcher will lead the session, with content validated by experts in plant protection and environmental health. It will involve PowerPoint presentations, visual aids, and interactive discussions on the following topics:

- **Introduction to Pesticides**: Definition, uses, and benefits.
- **Health and Environmental Risks**: An overview of the potential acute and chronic health effects of pesticide exposure and the environmental implications of pesticide misuse.
- **Pesticide Handling and Safety Practices**: This section covers best practices for handling pesticides, with a focus on the importance of PPE use and respect for preharvest intervals.
- **Understanding Pesticide Labels**: This is an explanation of the pictograms and color-coded hazard levels used on pesticide labels based on the Food and Agriculture Organization (FAO) and World Health Organization (WHO) guidelines.

Participants can ask questions and share their experiences with pesticide use. The session aims to create a supportive learning environment where farmers can engage with the material actively. Additionally, participants in the TESG group will be encouraged to apply what they’ve learned in their daily practices.

**Educational Video**

The Digital Video-Based Learning Group (DVBLG) will receive an educational video via WhatsApp. The video is approximately 4 minutes long and covers the same core content as the traditional educational session. Key features of the video include:

- **Concise Content**: The video presents information on pesticide risks, safe handling practices, label interpretation, and PPE usage in a straightforward and easily digestible format.
- **Visual Aids**: Animated visual aids and examples are incorporated to help farmers understand complex concepts, such as interpreting pesticide pictograms and color codes, enhancing their retention of the information.
- **Arabic voice-over**: The video includes a clear and engaging Arabic voice-over, ensuring it is accessible to all participants.
- **Reinforcement Mechanisms**: The short length and availability via WhatsApp allow participants to re-watch the video as needed, reinforcing their understanding over time.

A multidisciplinary team, including experts in agricultural safety and public health, developed and reviewed the video content to ensure it is relevant and culturally appropriate for the target audience.

**9. Timeline:**

| **Time point** | **Study period** | **Enrolment** | **Allocation** | **Intervention** | **Post- Intervention Assessment** |
| --- | --- | --- | --- | --- | --- |
| **June 10- June 30, 2024** | Recruitment | Eligibility screening |  |  |  |
| **July 1- July 21, 2024** | Baseline Assessment | Informed consent, Baseline data collection | Randomization |  |  |
| **July 22- August 9, 2024** | Intervention and Assessment |  |  | Intervention sessions (TESG and DVBLG) | Post-intervention assessment |

**10. Outcomes**

**Primary Outcome Measures**:

- Ability to correctly identify and interpret FAO pictograms on pesticide labels.
- Knowledge of health risks associated with pesticide use.
- Understanding of the environmental consequences of pesticide misuse.

**Secondary Outcome Measures**:

- Awareness of the importance of personal protective equipment (PPE) usage.
- Reported changes in pesticide handling practices and PPE compliance.

**11. Data Collection**

Data will be collected at two time points through face to face interview:

1. Baseline Assessment: Before the intervention, all participants will complete a structured questionnaire assessing their knowledge, practices, and understanding of pesticide safety labels.
2. Post-Intervention Assessment: Following the interventions, participants will complete a post-intervention questionnaire to measure changes in their knowledge and practices.

**12. Statistical Analysis**

The statistical analysis for this study will be conducted using the Statistical Package for the Social Sciences (SPSS; Chicago, IL, USA), version 25, with statistical significance set at p<0.05p < 0.05p<0.05.

Descriptive Analysis

The baseline characteristics of the participants across the three groups—Control Group (CG), Digital Video-Based Learning Group (DVBLG), and Traditional Educational Session Group (TESG)—will be summarized using descriptive statistics. For continuous variables, such as age and years of experience, we will calculate the mean and standard deviation (SD). For categorical variables, such as gender and prior pesticide training, frequencies and percentages will be presented.

Baseline Comparison

To confirm the comparability of the groups before the intervention, baseline characteristics will be assessed using a one-way analysis of variance (ANOVA) across the three groups, ensuring no significant differences could affect outcome interpretation.

Primary Outcome Analysis (Per-Protocol)

A per-protocol approach will be applied to analyze the primary outcomes, which include knowledge of pesticide safety, interpretation of pesticide labels, and understanding of the environmental impacts of pesticide misuse. The analysis will proceed as follows:

- Within-Group Changes: Paired t-tests will assess changes from baseline to post-intervention for each group.
- Between-Group Differences: A one-way ANOVA will compare differences between the groups in post-intervention scores. If significant differences are detected, post hoc tests (e.g., Tukey’s HSD) will be conducted to explore pairwise comparisons between groups.

Secondary Outcome Analysis

For secondary outcomes, including PPE usage and pesticide-handling practices, changes within each group will be assessed using paired t-tests, while differences between groups will be analyzed using one-way ANOVA with post hoc tests for further analysis as needed.

**12. Handling Missing Data**

Given the face-to-face interview method used to facilitate data entry through Google Docs, missing data is expected to be minimal. However, list-wise deletion will be applied to any remaining incomplete cases to ensure consistency across analyses. This strategy is intended to maintain accuracy while minimizing the impact of missing values on overall findings.

This approach will comprehensively assess intervention effectiveness across comparable groups, consistent with the analysis of the detailed results.

**13. Dissemination of Results**

Study results will be disseminated through publication in peer-reviewed journals and presentations at relevant conferences. A summary of findings will also be shared with the farmers who participated in the study.

**14. Conclusion**

This protocol outlines a rigorous approach to evaluating the effectiveness of WhatsApp-delivered video education in improving farmers' knowledge of pesticide safety and label interpretation. The findings from this study are expected to contribute to the development of scalable and cost-effective educational interventions in resource-limited settings.

**References.**

Abou Ibrahim, S., Naji, R., Zeineldeen, H., & Ghach, W. (2023). Effectiveness of pesticide labels (pictograms and color codes): A cross-sectional study of farmers’ understanding and practices in Lebanon. *Human and Ecological Risk Assessment: An International Journal*, 1–16. https://doi.org/10.1080/10807039.2023.2266036

Abou Zeid, M. I., Jammoul, A. M., Melki, K. C., Abou Jawdah, Y., & Awad, M. K. (2020). Suggested policy and legislation reforms to reduce deleterious effect of pesticides in Lebanon. *Heliyon*, *6*(12), e05524. https://doi.org/10.1016/j.heliyon.2020.e05524

Damalas, C. A. (2009). Understanding benefits and risks of pesticide use. *Sci. Res. Essays*, *4*(10), 945–949.

Dugger-Webster, A., & LePrevost, C. E. (2018). Following pesticide labels: A continued journey toward user comprehension and safe use. *Current Opinion in Environmental Science & Health*, *4*, 19–26.

Emery, S., Hart, A., Butler-Ellis, C., Gerritsen-Ebben, M., Machera, K., Spanoghe, P., & Frewer, L. (2015). A review of the use of pictograms for communicating pesticide hazards and safety instructions: Implications for EU policy. *Human and Ecological Risk Assessment: An International Journal*, *21*(4), 1062–1080.

Gevao, B., Kurt-Karakus, P. B., Birgul, A., Martinez-Guijarro, K., Sukhn, C., Krishnan, D., Rajagopalan, S., Hajeyah, M., Bahloul, M., & Alshemmari, H. (2022). Ambient air concentrations and risk assessment of selected organochlorine pesticides (OCPs) across five Middle Eastern countries. *Journal of Environmental Exposure Assessment*, *1*(3), 14. https://dx.doi.org/10.20517/jeea.2022.05

Handford, C. E., Elliott, C. T., & Campbell, K. (2015). A review of the global pesticide legislation and the scale of challenge in reaching the global harmonization of food safety standards. *Integrated Environmental Assessment and Management*, *11*(4), 525–536. https://doi.org/10.1002/ieam.1635

Maddah, D., Ghach, W., Abi Farraj, N., Yehya, M., Al Khatib, J., & Alami, N. H. (2020). The first community-based intervention to promote safe pesticide use by developing knowledge, attitudes, and practices among Lebanese farmers. *Human and Ecological Risk Assessment: An International Journal*, *26*(10), 2824–2835. https://doi.org/10.1080/10807039.2019.1688639

Mardigian, P., Chalak, A., Fares, S., Parpia, A., El Asmar, K., & Habib, R. R. (2021). Pesticide practices in coastal agricultural farms of Lebanon. *International Journal of Environmental Health Research*, *31*(2), 132–147. https://doi.org/10.1080/09603123.2019.1634797

MHEIDLY, N., GARCÍA GRAÑA, G., & SAMY TAYIE, S. (2021). LEARNING THROUGH WHATSAPP DURING THE COVID-19 PANDEMIC: A CASE STUDY. *Asia-Pacific Journal of Information Technology & Multimedia*, *10*(2).

Mostafalou, S., & Abdollahi, M. (2017). Pesticides: An update of human exposure and toxicity. *Archives of Toxicology*, *91*(2), 549–599.

Nicolopoulou-Stamati, P., Maipas, S., Kotampasi, C., Stamatis, P., & Hens, L. (2016). Chemical pesticides and human health: The urgent need for a new concept in agriculture. *Frontiers in Public Health*, *4*, 148. https://doi.org/10.3389/fpubh.2016.00148

Syafrudin, M., Kristanti, R. A., Yuniarto, A., Hadibarata, T., Rhee, J., Al-Onazi, W. A., Algarni, T. S., Almarri, A. H., & Al-Mohaimeed, A. M. (2021). Pesticides in drinking water—A review. *International Journal of Environmental Research and Public Health*, *18*(2), 468. https://doi.org/10.3390/ijerph18020468

Tsai, W.-T. (2013). A review on environmental exposure and health risks of herbicide paraquat. *Toxicological & Environmental Chemistry*, *95*(2), 197–206.

Waichman, A. V., Eve, E., & da Silva Nina, N. C. (2007). Do farmers understand the information displayed on pesticide product labels? A key question to reduce pesticides exposure and risk of poisoning in the Brazilian Amazon. *Crop Protection*, *26*(4), 576–583.

World Health Organization. (2022). *International Code of Conduct on Pesticide Management: Guidance on good labelling practice for pesticides (second revision)*. World Health Organization.
